# Covered or Uncovered

**Published:** 1892-08

**Authors:** John Dike

**Affiliations:** Melrose, Mass.


### COVERED OR UNCOVERED?

(*From Guiding Symptoms.*)

BY JOHN DIKE, M. D., MELROSE, MASS.

**BETTER FROM COVERING.**—Acon., Aman., Am-m., Anthro., Ant-c., Arg-met., Arg-nit., Arn., Ars., Ars-hyd., Asim-tr., Ast-fl., Aur-met., Bell., Benz-ac., Caps., Caust., China, Clem., Coff., Corr-r., Cycl., Eup-perf., Gels., Hell., Hep., Hippom., Kali-bi., Mag-c., Mag-m., Natr-m., Nitr-ac., Nux-mos., Nux-vom., Phos-ac., Phos., Psor., Rheum., Rhust., Rumex., Samb., Sep., Sil., Squill., Stram., Stront-c., Syph., Thuj.

**BETTER FROM UNCOVERING.**—Amyl-nit., August., Apis., Ars-met. [feet], Ast-rub., Aur-mur., Borax, Bry., Calad., Calc-c., Camph., Cannab-s., Carb-veg., Chin-ars., China, Corr-r., Ferr., Fluor-ac., Hyos., Ig., Iodoform, Iod., Jatroph., Led., Lyc., Manc., Medorr., Merc., Mosc., Mur-ac., Natr-c., Nux-v., Op., Phos. [scalp], Puls., Sang., Sc-c., Spig., Staph., Sulph., Zn.

